# Interplay between negative symptoms, time spent doing nothing, and negative emotions in patients with schizophrenia spectrum disorders: results from a 37-site study

**DOI:** 10.1038/s41537-023-00372-x

**Published:** 2023-09-21

**Authors:** Giulio D’Anna, Cristina Zarbo, Giuseppe Cardamone, Manuel Zamparini, Stefano Calza, Matteo Rota, Christoph U. Correll, Matteo Rocchetti, Fabrizio Starace, Giovanni de Girolamo, DIAPASON collaborators, Stefano Barlati, Andrea Baroncelli, Filippo Besana, Maria Elena Boero, Massimo Clerici, Vittorio Di Michele, Federico Durbano, Maria Gabriella Foia, Stefania Impicci, Filippo Maria Jacoponi, Emanuela Leuci, Daniela Malagamba, Marina Marina, Alessandra Martinelli, Emiliano Monzani, Gaetano Nappi, Benedetto Piccicacchi, Roberto Placenti, Arturo Rippa, Giambattista Tura, Salvatore Zizolfi

**Affiliations:** 1 Department of Mental Health, AUSL Toscana Centro, Prato, Italy; 2 Department of Psychology, University of Milano Bicocca, Milan, Italy; 3 Unit of Epidemiological and Evaluation Psychiatry, IRCCS Istituto Centro San Giovanni di Dio Fatebenefratelli, Brescia, Italy; 4 Unit of Biostatistics and Bioinformatics, Department of Molecular and Translational Medicine, University of Brescia, Brescia, Italy; 5 Department of Psychiatry, The Zucker Hillside Hospital, Northwell Health, Glen Oaks, NY USA; 6 Department of Psychiatry and Molecular Medicine, Donald and Barbara Zucker School of Medicine at Hofstra/Northwell, Hempstead, NY USA; 7 Department of Child and Adolescent Psychiatry, Charité Universitätsmedizin Berlin, Berlin, Germany; 8 Department of Mental Health and Dependence, ASST of Pavia, Pavia, Italy; 9 Department of Mental Health and Pathological Addiction, AUSL di Modena, Modena, Italy; 10 DSMD, ASST Spedali Civili, Brescia, Italy; 11 DSM, USL Toscana Centro, Prato, Italy; 12 DSMD, ASST di Pavia, Pavia, Italy; 13 Fatebenefratelli Beata Vergine Consolata, San Maurizio Canavese, Torino Italy; 14 DSMD, ASST Monza, Monza, Italy; 15 DSM, ASL Pescara, Penne, Pescara Italy; 16 DSMD, ASST Melegnano and Martesana, Melegnano, Italy; 17 DSM, Napoli 2 Nord, Frattamaggiore, Napoli Italy; 18 DSM, ASL Ancona, Ancona, Italy; 19 Passaggi Srl-Oricola, Aquila, Italy; 20 DSMD, AUSL Parma, Parma, Italy; 21 DSM, ASL 3 Genova, Genoa, Italy; 22 Fondazione Giuseppe Costantino, Pavia, Italy; 23 DSM, AOUI Verona; IRCCS Fatebenefratelli San Giovanni di Dio, Brescia, Italy; 24 DSMD, ASST Bergamo Ovest, Treviglio, Bergamo Italy; 25 DSM, ASL Bari, Bari, Italy; 26 Centro Ippocrate, Cagliari, Italy; 27 Centro Sacro Cuore Di Gesù FBF, San Colombano Al Lambro, Milano Italy; 28 DSM, ASUITS Trieste, Trieste, Italy; 29 IRCCS Fatebenefratelli, Brescia, Italy; 30 CREST “La Perla”, Grumello del Monte, Bergamo, Italy

**Keywords:** Schizophrenia, Human behaviour

## Abstract

This study evaluated the relationship between negative symptoms, daily time use (productive/non-productive activities, PA/NPA), and negative emotions in schizophrenia-spectrum disorders (SSDs): 618 individuals with SSDs (311 residential care patients [RCPs], 307 outpatients) were surveyed about socio-demographic, clinical (BPRS, BNSS) and daily time use (paper-and-pencil Time Use Survey completed twice/week) characteristics. Among them 57 RCPs and 46 outpatients, matched to 112 healthy controls, also underwent ecological monitoring of emotions (8 times/day for a week) through Experience Sampling Method (ESM). RCPs spent significantly less time in PA than outpatients. Patients with more negative symptomatology spent more time in NPA and less in PA compared to patients with milder symptoms. Higher time spent in NPA was associated with negative emotions (*p* < 0.001 during workdays) even when correcting for BNSS total and antipsychotic polypharmacy (*p* = 0.002 for workdays, *p* = 0.006 for Sundays). Future studies are needed to explore in more detail the relationship between negative emotions, negative symptoms, time use, and functioning in individuals with SSDs, providing opportunities for more informed and personalised clinical treatment planning and research into interactions between different motivational, saliency and behavioural aspects in individuals with SSDs.

## Introduction

Schizophrenia-spectrum disorders (SSD) are severe and chronic conditions that are associated with significant functional impairment^1^. Negative symptoms (i.e., anhedonia, asociality, avolition, blunted affect, alogia) have long been acknowledged to play a key role in the clinical expression and related disability of SSDs^2^. In recent years, the definition of this multi-faceted cluster of psychopathological dimensions has been widely explored – mostly by highlighting its putative core domains^3–5^, and the complex relationship between primary and secondary negative symptoms^6,7^. Secondary negative symptoms constitute a significant clinical challenge, as they are largely determined by certain side effects of antipsychotic medications, including extrapyramidal side effects and sedation^8^, and lack of stimulation within the psychosocial environment^9^. A further underpinning of secondary negative symptoms is the frequent co-occurrence of depressive symptoms^10^, including low mood and negative beliefs, self-neglect, and sedentary behaviour. This complex framework leads to poor personal functioning, greater severity of symptoms, unemployment or other forms of reduced social engagement, and inactive lifestyle – thus resulting in worse clinical outcomes^11^, and increased physical morbidity and mortality^12,13^.

On the one hand, the inner experiences and behavioural features that contribute to defining negative symptoms have been associated with time spent in passive activities^14,15^, reduced social inclusion^16,17^, and lower engagement in active lifestyle^18,19^. Furthermore, factors such as antipsychotic treatment, unemployment, and low environmental stimulation in daily life settings may worsen the burden of negative symptoms and lack of productive time use^20^. The longitudinal importance of these intertwined aspects is confirmed by the fact that vocational inactivity is tied to poor prognosis in first-episode psychosis^21^.

In terms of treatment implications, a higher burden of negative symptoms may act as a maintaining factor through a reduced impact of lifestyle interventions^22^. In this complex framework, negative emotions and demotivating beliefs may also contribute to the perpetuation of inactivity and social withdrawal^23^, and therefore to reduced engagement in psychosocial initiatives, warranting investigation in real-world environments. In fact, even if the reliability of emotion expression and assessment in people with SSD is debated^24,25^, it has been suggested that experiencing negative emotions may represent a predictor of reduced subjective recovery^26^. Moreover, from a cognitive perspective, attitudinal and motivational factors intertwined with negative emotions may play a key role in the poor functioning of people with SSD^27–29^, especially in patients’ daily experience^30,31^. For these reasons, the investigation of negative emotions may enrich the established neurocognitive paradigm of negative symptoms and related functional outcomes.

Even though severe and persistent mental disorders are frequently tied to an imbalance in daily time use^32,33^, research on SSD has been mostly conducted in the context of occupational interventions^16,34–36^. However, the recent increase of observational studies based on the Experience Sampling Method (ESM) is more likely to shed light on real-world experiences of patients with severe psychosis^37^. In fact, several ESM studies have proved its reliability and validity for different objectives, populations, and settings^38^. Eventually, this gain in knowledge may lead to an improvement in the multi-modal treatment of negative symptoms^39^.

In the context of the multicenter DiAPAson study, which aims to understand the daily experience of outpatients and residential care patients suffering from SSDs^40^, this study explored the complex relationship between negative symptoms, inactivity (i.e., time spent in non-productive activities, NPA), and negative emotions. Specifically, we aimed to: (1) evaluate differences in the amount of time spent in NPA and productive activities (PA) of both work days and Sundays among residential care patients, outpatients, and healthy controls; (2) examine the relationship between negative symptoms and time spent in NPA and PA; and (3) test the influence of the amount of NPA on negative emotions – adjusting for negative symptomatology severity and antipsychotic polypharmacy as a marker of increased postsynaptic dopamine blockade and potential secondary negative symptoms^3,41^, in a subsample of patients who underwent ecological monitoring assessments with ESM. We hypothesised that patients would spend more time in NPA and less time in PA than healthy controls, and that more negative symptomatology would be associated with more time spent in NPA and less time in PA. Moreover, we hypothesised that less NPA would be associated with more negative daily negative emotions, over and above the adverse effect of negative symptomatology severity and antipsychotic polypharmacy.

## Methods

### Study setting and procedures

The DiAPASon multisite project (DAily time use, Physical Activity, quality of care, and interpersonal relationships in patients with Schizophrenia spectrum disorders) involved 20 Departments of Mental Health (DMHs) and 17 residential facilities (RFs) located in various regions of Italy. The participating DMHs recruited both outpatients and residential patients, while RFs only enroled residential care patients. A total of 98 RFs participated, with an average number of 12.8 (±5.7) residents, which together recruited an average of 3.5 (±2.6) patients per RF (approximately 27% of patients in each RF).

At each study centre, treating clinicians invited eligible patients under their care to participate in the study. To reduce selection bias, outpatients (who were community-dwelling patients with SSDs) were recruited from consecutively evaluated patients at the DMHs until the desired target sample was achieved. Similarly, in the RFs, facility directors prepared an alphabetical list of patients with SSDs who were present on an index day: based on this list, patients were consecutively invited to participate in the study until the required target sample was achieved. Healthy controls were recruited by public advertisement and snowball sampling procedures.

Participants received detailed information about the study and had the opportunity to ask questions. Socio-demographic and clinical data were collected by treating clinicians, while research assistants (RAs) helped the patients complete a range of self-report questionnaires and the time use survey (TUS) – see sections below and the study protocol for details^40^. Subsequently, participants from a selected number of sites were invited to take part to the ESM monitoring; logistic and financial limitations prevented the implementation of the ESM study in all sites. The ESM monitoring was preceded by a briefing session in which RAs gave instructions about the procedures and how to effectively perform them. The monitoring was followed by a debriefing session in which the same RAs collected information on study acceptability and feasibility. During the debriefing session, outpatients and healthy controls received € 25.00 for travel expense reimbursement.

The study was conducted in accordance with the American Psychological Association^42^ ethical standards for the treatment of human experimental volunteers. All participants provided written informed consent in compliance with the Declaration of Helsinki^43^. The study was approved by the ethical committee of the local institutions (see section below).

### Participants

The study involved patients with a SSD diagnosis according to the Diagnostic and Statistical Manual of Mental Disorders, Fifth Edition (DSM-5) criteria^44^, aged 20–55, able to talk and write in Italian, and treated at RFs or as outpatients at the DMH. To minimise selection biases, the following exclusion criteria were applied: inability to provide informed consent, presence of a severe cognitive deficit (i.e., a Mini-Mental State Examination – MMSE – corrected score of less than 24), recent (last 6 months) diagnosis of substance use disorder according to DSM-5 criteria^44^, or history of clinically significant head injury or cerebrovascular/neurological disease.

### Instruments

#### Clinical assessments

Psychiatric history was assessed using a structured ad hoc survey to collect information on current diagnosis and treatments, illness duration, and lifetime duration of psychiatric hospitalisation. Negative symptom severity was assessed using the Brief Negative Symptom Scale (BNSS)^45,46^, a 13-item clinician-administered instrument designed to evaluate anhedonia, distress related to negative symptoms, asociality, avolition, blunted affect, and alogia. Each subscale is rated on a scale from 0 (not present) to 6 (severe deficit), with higher values indicating greater symptom severity.

#### Paper-and-pencil Time Use Survey (TUS)

Daily activity refers to the range of behaviours and actions individuals engage in throughout their daily lives: EUROSTAT, for their Harmonised European Time Use Surveys (HETUS)^47^, has identified 146 categories of daily activities; we collapsed these 146 detailed activities into 15 broad TUS categories (Supplementary Table 1S shows all activities listed in the TUS questionnaire and the sub-categories they include).

In the TUS questionnaire, each column indicated the daily hour (from 12 a.m. to 11.59 p.m.); for any daily hour, each participant had to answer the question “*What are you doing right now?*”, selecting one or more of the 15 possible activity categories. The TUS was completed by both people with SSD and the unaffected control group, and each selection in the TUS corresponded to a “count” of about 60 min. The TUS was completed by each participant twice during a week, on a working day (Monday-Friday) and on Sunday. The participants were invited to complete the survey at different times during the day or at latest in the evening, in order to minimise recall biases as much as possible.

In this study, we focused on NPA and PA only and considered data collected from 7 a.m. to 11.59 p.m., as we were interested in the relationship between PA/NPA and emotions, which were assessed only during this time frame in the ESM study (see below). In particular, we considered five ‘Productive Activities’ (PA) which are goal-oriented, purposeful, and contribute to personal growth, skill development, or achievement and include “working”, “studying”, “doing housework”, “taking care of someone or something” and “voluntary work”; Non-Productive Activities (NPA) are typically characterised by a lack of clear goals or direct contribution to personal growth; they may involve passive or unproductive behaviours and include “sleeping”, “staying in bed” and “resting, doing nothing”. This categorisation, based on EUROSTAT time use surveys, was built in accordance with the main goals of the research project^40^. For example, the NPA macro-category did not include *“watching TV or listening to the radio”* to separate those activities that can be considered entirely passive (e.g., doing nothing) and those that may involve an active cognitive effort (i.e., watching a documentary, following a storyline plot in a movie, etc.).

#### Assessment of daily emotions with ESM

Daily emotions were assessed with a brief questionnaire on a smartphone-based application for ESM which was developed ad hoc for the project. The ESM assessment included three sections: current activities, social contacts, and emotions.

The emotions section randomly presented seven emotions (i.e., happy, sad, tired, relaxed, nervous, quiet, full of energy) that have been included, based on previous studies, in an ESM item repository (https://osf.io/kg376/wiki/home/), as suggested by Myin-Germeys and Kuppens^48^. The main objective of this repository is to collect items used in several ESM studies in order to facilitate the selection of good assessment measures and the exchange of researchers’ experiences. In our research project, this section asked the participant how he/she felt that emotion at that current moment. The participant had to select the amount of that emotion on a visual bar from 0 to 100 (from 0, *“not at all”* to 100, *“a lot”*). Positive emotions were computed as mean of the following items: happy, relaxed, quiet, full of energy. Negative emotions, which were the focus of this study, were computed as mean of the following ESM items: sad, tired, nervous. Emotions were calculated for both weekdays and weekends. Participants received notifications 8 times a day, from 8 a.m. to 11:59 p.m., for 7 consecutive days. The notifications were semi-randomised (i.e., randomly sent within eight scheduled time slots), and a reminder was sent after 15 minutes if the participant did not respond to the initial prompt. Participants had a maximum of 30 min to respond.

### Statistical analyses

Descriptive statistics consisted of frequency tables for categorical variables and means (±standard deviations) for continuous variables. All variables were analysed using linear models, with a log-transformation for BNSS Total, BNSS Distress, BNSS Blunted affect, and BNSS Alogia scores, as well as PA. The comparison of categorical variables across groups of residential care patients, outpatients, and controls was performed with the Chi-square test with *p-values* computed via Monte-Carlo simulation (B = 2000). Correlations between BNSS domains and time spent in NPA and PA were expressed as Pearson’s correlations. Among patients, three groups based on negative symptomatology severity (created by tertiles of mean BNSS total scores) were compared: Low Negative Symptom Severity (LNSS); Medium Negative Symptom Severity (MNSS); High Negative Symptom Severity (HNSS). Pairwise *post hoc* comparisons p-values were adjusted using the Holm–Bonferroni procedure^49^. The association between negative emotions and the amount of NPA was adjusted for BNSS total score (due to the trend-level significant univariate association between negative emotions and BNSS total score) and antipsychotic polypharmacy (i.e., the use of 2 or more antipsychotics drugs, due to likely difference in the overall chlorpromazine equivalent dose of antipsychotic drugs and related secondary negative symptoms between the two groups^41^). Supplementary Tables 6S–10S report the values for test statistics used throughout the main text. All tests were two-sided and assumed a 5% significance level. The version 27.0 of the SPSS software^50^ was used for statistical analyses.

## Results

### Patient flow

From October 2020 to October 2021, 673 eligible patients (340 residential patients, 333 outpatients) and 114 healthy controls were recruited. Among the 673 patients initially selected, 17 residential patients (2.5%) were excluded for severe cognitive impairment (i.e., MMSE < 24), 36 patients (10 residential patients and 26 outpatients, 5.3%) dropped out from the study after providing initial consent to participate, whereas 2 residential patients and 2 healthy controls did not complete the TUS. Therefore, the final sample for the first (paper-and-pencil) phase of this study included 618 patients (311 residential care patients, 307 outpatients) and 112 healthy controls. Altogether, 121 patients (66 residential care patients, 55 outpatients) and 112 healthy controls (matched for age and sex) participated in the TUS and the ESM.

### Socio-demographic and clinical characteristics of the sample

Table 1 shows the socio-demographic and clinical characteristics of the sample, and between-group differences. Groups were homogeneous regarding sex distribution and mean age. Both residential care patients and outpatients were more frequently single (86.8% and 85.7%, respectively) compared to healthy controls (25.0%), who, in turn, had a higher mean education level and were more likely to work compared to both patient groups. Unemployment was more common among residential care patients compared to outpatients. Outpatients (28.6 ± 6.0) had a higher body mass index (BMI) compared to both residential patients (26.9 ± 4.9; *p* < .001) and healthy controls (24.2 ± 3.7; *p* < .001), and residential care patients had a higher BMI compared to controls (*p* < .001).

**Table 1:** Socio-demographic data, clinical features and time use in participants with schizophrenia-spectrum disorders and healthy controls.

					Residential care vs outpatients		Residential care vs controls		Outpatients vs controls	
Characteristic	Residential care patients (*n* = 311)	Outpatients (*n* = 307)	Healthy controls (*n* = 112)	*p*	Mean difference (*CI* 95%)	*p*	Mean difference (*CI* 95%)	*p*	Mean difference (*CI* 95%)	*p*
Socio-demographic information										
Age (M ± SD)	41.0 (9.8)	41.7 (9.2)	41.4 (10.2)	0.737	−	−	−	−	−	−
Sex (*n* males, %)	218 (70.1%)	202 (65.8%)	67 (59.8%)	0.128	−	−	−	−	−	−
Marital status (*n*, %)										
Divorced/widowed	28 (9.0%)	14 (4.6%)	6 (5.4%)	**<0.001**	**−**	**0.003**	**−**	**<0.001**	**−**	**<0.001**
Married/cohabiting	13 (4.2%)	30 (9.8%)	78 (69.6%)							
Single	270 (86.8%)	263 (85.7%)	28 (25.0%)							
Education years (M ± SD)	11.5 (3.2)	11.9 (3.0)	16.5 (4.9)	**<0.001**	−0.4 (−0.9; 0.0)	0.074	−5.1 (−6.0; −4.2)	**<0.001**	−4.6 (−5.5; −3.8)	**<0.001**
Working status (*n*, %)										
Working	38 (12.2%)	90 (29.3%)	103 (92.0%)	**<0.001**	**−**	**<0.001**	**−**	**<0.001**	**−**	**<0.001**
Studying	14 (4.5%)	21 (6.8%)	8 (7.1%)							
Not working nor studying	259 (83.3%)	196 (63.8%)	1 (0.9%)							
Body Mass Index (M ± SD)	26.9 (4.9)	28.6 (6.0)	24.2 (3.7)	**<0.001**	−1.7 (−2.6; −0.9)	**<0.001**	2.7 (1.4; 4.1)	**<0.001**	4.5 (3.1; 5.8)	**<0.001**
Psychopathology										
SSD diagnosis (*n*, %)										
Delusional Disorder	15 (4.8%)	8 (2.6%)	−	**0.018**^a^	−	−	−	−	−	−
Schizophreniform Disorder	3 (1.0%)	5 (1.6%)	−							
Schizophrenia	160 (51.5%)	196 (63.8%)	−							
Schizoaffective Disorder	76 (24.4%)	58 (18.9%)	−							
Other SSD (i.e., without specification, with other specification)	57 (18.3%)	40 (13.0%)	−							
Psychiatric comorbidities (*n* yes, %)	112 (36.0%)	64 (20.9%)	−	**<0.001**	−	−	−	−	−	−
Illness duration (years, M ± SD)	18.3 (9.6)	18.1 (9.4)	−	0.856	−	−	−	−	−	−
Antipsychotic drugs (M ± SD)	1.8 (0.8)	1.4 (0.7)	−	**<0.001**	0.4 (0.2; 0.5)	−	−	−	−	−
Non-antipsychotic psychotropic drugs (M ± SD)	1.6 (1.2)	1.0 (1.1)	−	**<0.001**	0.6 (0.5; 0.8)	−	−	−	−	−
Antidepressants	0.3 (0.5)	0.3 (0.5)	−	0.406	−	−	−	−	−	−
Benzodiazepines	0.9 (0.8)	0.4 (0.6)	−	**<0.001**	0.5 (0.4; 0.6)	−	−	−	−	−
Mood stabilisers	0.4 (0.6)	0.2 (0.5)	−	**<0.001**	0.2 (0.1; 0.2)	−	−	−	−	−
BNSS total score^b^ (M ± SD)	26.3 (16.6)	19.3 (13.9)	−	**<0.001**	6.9 (4.5; 9.4)	−	−	−	−	−
Anhedonia	2.1 (1.6)	1.5 (1.4)	−	**<0.001**	0.5 (0.3; 0.8)	−	−	−	−	−
Distress^b^	2.2 (1.8)	1.4 (1.5)	−	**<0.001**	0.8 (0.5; 1.0)	−	−	−	−	−
Asociality	2.2 (1.5)	1.9 (1.5)	−	0.**003**	0.4 (0.1; 0.6)	−	−	−	−	−
Avolition	2.1 (1.6)	1.7 (1.4)	−	**<0.001**	0.4 (0.2; 0.7)	−	−	−	−	−
Blunted affect^b^	1.9 (1.6)	1.3 (1.4)	−	**<0.001**	0.6 (0.3; 0.8)	−	−	−	−	−
Alogia^b^	1.7 (1.7)	1.1 (1.4)	−	**<0.001**	0.6 (0.4; 0.8)	−	−	−	−	−
Daily activities from time use survey (count frequency)										
NPA on Sundays (M ± SD)	5.8 (2.8)	5.5 (3.1)	3.1 (2.0)	**<0.001**	0.3 (−0.3; 0.8)	0.459	2.7 (2.0; 3.4)	**<0.001**	2.4	**<0.001**
Median (range)	5 (0–20)	5 (0–17)	3 (0–10)						(1.7; 3.2)	
NPA on work days (M ± SD)	4.9 (2.6)	4.5 (3.1)	1.1 (1.2)	**<0.001**	0.5 (0.0; 0.9)	0.091	3.8 (3.1; 4.5)	**<0.001**	3.4	**<0.001**
Median (range)	4 (0–15)	4 (0–19)	1 (0–7)						(2.7; 4.1)	
PA on Sundays (M ± SD)^b^	1.8 (2.3)	2.9 (3.1)	5.3 (4.2)	**<0.001**	−1.1 (−1.5; −0.6)	**<0.001**	−3.5 (−4.3; −2.7)	**<0.001**	−2.4	**<0.001**
Median (range)	1 (0–15)	2 (0–18)	4 (0–17)						(−3.2; −1.7)	
PA on work days (M ± SD)^b^	2.4 (2.4)	4.4 (3.3)	11.1 (3.8)	**<0.001**	−2.1 (−2.7; −1.5)	**<0.001**	−8.7 (−9.5; −7.9)	**<0.001**	−6.6	**<0.001**
Median (range)	2 (0–12)	4 (0–16)	11 (0–28)						(−7.4; −5.8)	

*M* *±* *SD* mean ± standard deviation, *SSD* Schizophrenia Spectrum Disorder, *BNSS* Brief Negative Symptom Scale, *NPA* Non-Productive activities, *PA* Productive Activities.

^a^*p* computed using Monte Carlo-simulation (B = 2000).

^b^Variable modelled on log scale.

Significant *p*-values are showed in bold.

Residential care patients had a higher frequency of psychiatric comorbidities (36.0% vs 20.9%; *p* < 0.001) and received a higher number of current antipsychotic (1.8 ± 0.8 vs 1.4 ± 0.7; *p* < 0.001) and non-antipsychotic (1.6 ± 1.2 vs 1.0 ± 1.1; *p* < 0.001) medications compared to outpatients. Residential care patients also had significantly higher BNSS total scores (26.3 ± 16.6 vs 19.3 ± 13.9; *p* < 0.001) and sub-scores than outpatients.

We also shown the socio-demographic and clinical characteristics of the ESM sample, and between-group differences (Supplementary Table 5s).

### Time use differences between patients and controls

Table 1 also shows between-group differences for daily time use, measured as daily frequency of NPA or PA. We did not find any statistically significant difference between outpatients and residential care patients regarding the amount of time spent in NPA (*p* = 0.459 for Sundays; *p* = 0.091 for work days). Conversely, outpatients spent significantly more time performing PA than residential care patients, both on work days (4.4 ± 3.3 vs 2.4 ± 2.4; *p* < 0.001) and on Sundays (2.9 ± 3.1 vs 1.8 ± 2.3; *p* < 0.001). Healthy controls spent less time in NPA, and more time in PA than both patient populations.

### Relationship between negative symptomatology and time use

As shown in Table 2, there were significant positive relationships between BNSS total scores, and the amount of time spent in NPA during working days – both among residential care patients (r = 0.18; *p* = 0.001) and outpatients (r = 0.13; *p* = 0.030). Conversely, non-significant negative relationships were found between BNSS total scores, and the amount of time spent in PA during work days – both among residential care patients (r = −0.11; *p* = 0.090) and outpatients (r = −0.10; *p* = 0.096). A significant relationship between BNSS total score and time use on Sundays was found only for residential care patients. Residential care patients showed similar trends for BNSS asociality, avolition and alogia subscales. On the other hand, outpatients mainly showed significant associations between time use during work days and the BNSS avolition subscale. The avolition subscale displayed the highest effect sizes (*r* ranging from −0.25 to 0.20) for both residential care and outpatients.

**Table 2:** Correlation matrix between negative symptoms and time use in residential care patients and outpatients during both work days and sundays.

	Residential patients (*N* = 311)								Outpatients (*N* = 307)							
	Number of non-productive activities during work days		Number of non-productive activities during Sundays		Number of productive activities during work days^b^		Number of productive activities during Sundays^b^		Number of non-productive activities during work days		Number of non-productive activities during Sundays		Number of productive activities during work days^b^		Number of productive activities during Sundays^b^	
	r	*p*	r	*p*	r	*p*	r	*p*	r	*p*	r	*p*	r	*p*	r	*p*
BNSS total score^b^	0.18	**0.001**^a^	0.17	**0.003**	−0.11	0.090	−0.16	**0.034**	0.13	**0.030**	0.09	0.120	−0.10	0.096	−0.12	0.073
Anhedonia	0.14	**0.014**	0.15	**0.007**	−0.09	0.114	−0.07	0.230	0.06	0.293	0.06	0.264	−0.07	0.209	−0.07	0.237
Distress^b^	0.14	**0.032**	0.19	**0.004**	−0.11	0.166	−0.20	**0.020**	0.01	0.930	−0.04	0.603	−0.09	0.273	0.08	0.391
Asociality	0.07	0.236	0.14	**0.014**	−0.14	**0.039**	−0.18	**0.011**	0.11	0.061	0.06	0.317	−0.04	0.498	−0.07	0.297
Avolition	0.19	**<0.001**^a^	0.17	0.**003**	−0.22	**<0.001**^a^	−0.25	**<0.001**^a^	0.20	**<0.001**^a^	0.09	0.116	−0.15	**0.013**	−0.09	0.196
Blunted affect^b^	−0.03	0.615	−0.05	0.427	0.05	0.548	−0.09	0.291	−0.05	0.437	0.01	0.866	0.04	0.588	0.02	0.795
Alogia^b^	0.16	**0.022**	0.05	0.480	−0.10	0.231	−0.21	**0.016**	−0.01	0.983	0.03	0.734	−0.11	0.213	−0.02	0.812

*BNSS* Brief Negative Symptom Scale.

^a^Still significant after Holm–Bonferroni correction for multiple testing.

^b^Variable log-transformed before Pearson’s correlation.

Significant *p*-values are showed in bold.

**Fig. 1:**
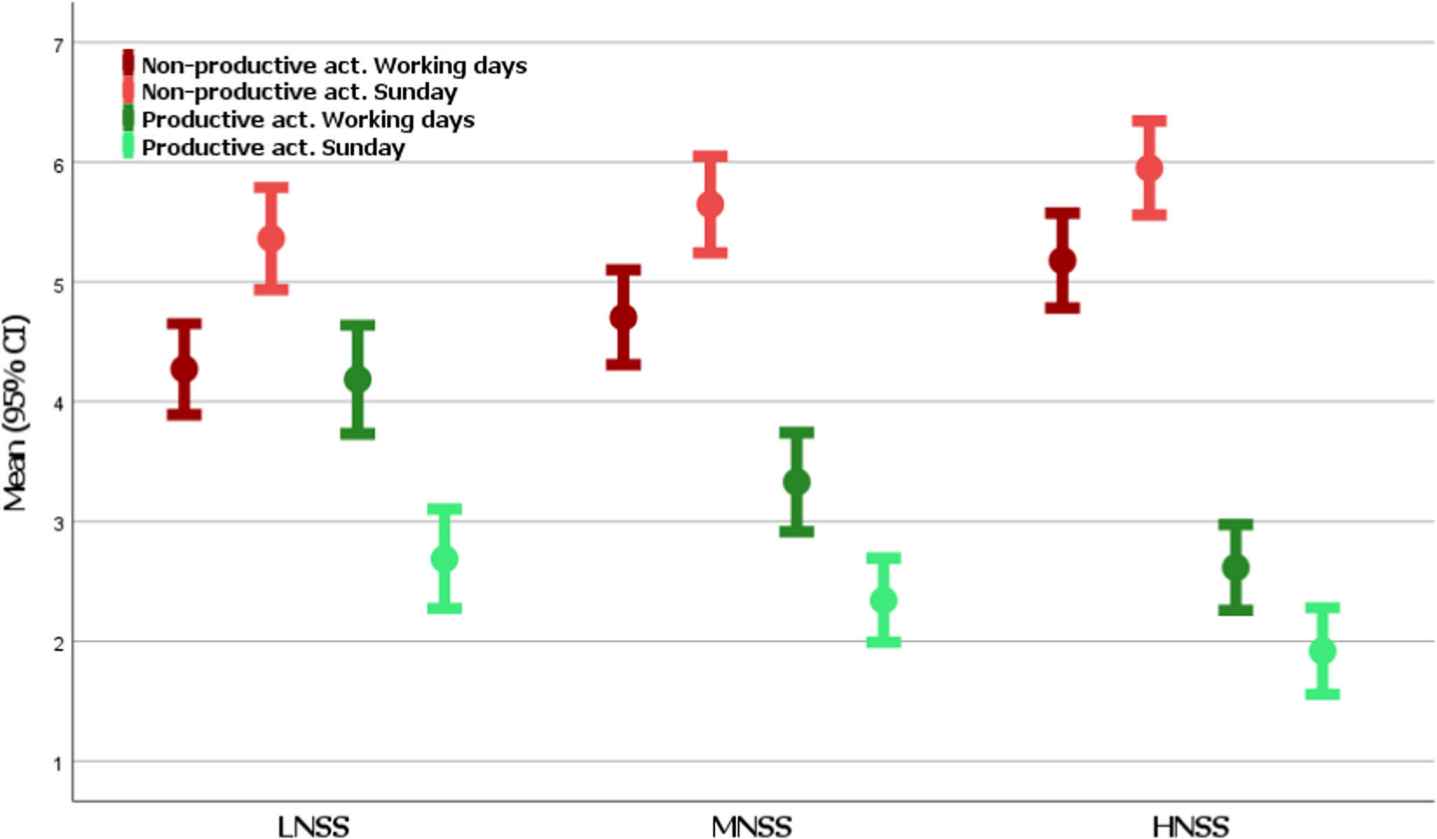
Differences in occasion of non-productive activities during workdays and during sundays for different negative symptom severity groups. LNSS Low Negative Symptom Severity, MNSS Medium Negative Symptom Severity, HNSS High Negative Symptom Severity.

In addition, between-group comparisons based on negative symptom severity identified differences between LNSS and HNSS subgroups (Fig. 1, Supplementary Table 2S) for the following variables: time spent in NPA on work days (4.3 ± 2.8 LNSS vs 5.2 ± 2.8 HNSS; *p* = 0.004) and time spent in productive activities on work days (4.2 ± 3.3 LNSS vs 2.6 ± 2.6 HNSS; *p* < 0.001).

### Relationship between non-productive activities, negative symptoms, and daily negative emotions assessed with ESM

We found that the BNSS total score showed a weak and marginally non-significative positive association with daily negative emotions assessed with ESM during work days ($\hat{\beta }$ = 0.178; *p* = 0.071), while this association was higher and statistically significant when considering only Sundays ($\hat{\beta }$ = 0.225; *p* = 0.029). Being in treatment with 2 or more antipsychotics also showed a significant positive association with daily negative emotions assessed with ESM during work days ($\hat{\beta }$ = 2.195; *p* = 0.046), and this association was higher when considering only Sundays ($\hat{\beta }$ = 4.193; *p* < 0.001). On the other hand, the amount of time spent in NPA was strongly associated with negative emotions ($\hat{\beta }$ = 1.389; *p* < 0.001 during work days, $\hat{\beta }$ = 1.512; *p* < 0.001 considering only Sundays) (Supplementary Tables 3–3S). The amount of time spent in NPA continued being significantly directly associated with daily negative emotions even when controlled for BNSS total scores ($\hat{\beta }$ = 1.681; *p* = 0.002 during work days, $\hat{\beta }$ = 1.385; *p* = 0.006 considering only Sundays) (Supplementary Tables 3–3S).

**Table 3:** Influence of non-productive activities on negative emotions in residential patients and outpatients.

Work days			
Variables	$\hat{\beta }$	*CI* (95%)	*p*
BNSS total score	0.151	−0.042; 0.345	0.124
Poly-AP treatment	−2.099	−7.473; 3.275	0.441
Non-productive activities	1.681	0.607; 2.756	0.002
Sundays			
Variables	$\hat{\beta }$	*CI* (95%)	*p*
BNSS total score	0.190	−0.010; 0.390	0.062
Poly-AP treatment	0.351	−5.310; 6.012	0.902
Non-productive activities	1.385	0.404; 2.365	0.006

*BNSS* Brief Negative Symptom Scale, *CI* confidence interval.

The association of NPA and negative emotions during a day (ESM) is weak and similar in the control group ($\hat{\beta }$ = 0.694; *p* = 0.155 during work days, $\hat{\beta }$ = 1.262; *p* = 0.139 considering only Sundays) and outpatients ($\hat{\beta }$ = 0.774; *p* = 0.259 during work days, $\hat{\beta }$ = 1.225; *p* = 0.118 considering only Sundays), while it is much more evident and statistically significant in residential patients ($\hat{\beta }$ = 2.081; *p* = 0.003 during work days, $\hat{\beta }$ = 2.017; *p* = 0.010 considering only Sundays) (Supplementary Table 4s).

## Discussion

The present study focused on the interplay of negative symptom severity, inactivity, and negative emotions in a large clinical sample of patients diagnosed with SSDs. Results revealed significant differences in daily time use between patients and controls, a significant direct association between non-productive daily time use and negative symptomatology in patients, and a notable direct association between NPA and negative emotions in this population, over and above negative symptomatology. The role of negative emotions for the functioning of individuals with SSDs is receiving increasing attention^24^. Even if it is debated whether SSD patients experience difficulties in emotional awareness – with significant implications for related self-reported assessments – negative emotions influence goal-directed behaviour and daily functioning^24,25^. For this reason, their investigation in real-world environments may improve therapeutic plans and can promote the development of cognitive strategies targeting motivational factors, dysfunctional beliefs, and related negative emotions. Putative psychological underpinnings of the functional consequences may be represented by discrepancies between the ideal and actual affect^51^, and anticipatory factors influencing motivation and related social functioning^52^. In particular, recent studies seem to show that negative emotions cannot be simply considered as the expression of stable, neurocognitive deficits which present as negative symptoms, but they deserve attention per se in light of the potential clinical implications^27–29^. ESM can significantly enrich the real-world assessment of objective behaviour and thereby inform both clinical care planning and the related research questions^30,31^.

### Patients spend more time in non-productive activities than healthy controls

We found that both patient groups spent more time in NPA than healthy controls. This finding should be considered in the context of the patients’ lower education level – an indirect information on illness onset and associated disability^53^. Moreover, the higher BMI may be a proxy of lower psychosocial adjustment^54^ and/or greater illness severity leading to more antipsychotic and other psychiatric medication use that can increase body weight^55^. Regarding time use, the imbalance in favour of NPA is common across a wide range of mental disorders^32,33^, and loss of productivity is a key outcome of interest when evaluating conditions associated with severe functional impairment^1^.

The present study confirms literature highlighting that negative symptoms are associated with a higher burden of time spent alone in passive activities^14–17^. This result held true both for individuals living in residential facilities and for outpatients. Shifting to clinical subgroups, residential care users reported being less engaged in PA, irrespective of the days considered. These findings indirectly reflect the differential degrees of autonomy at different intensities and settings of psychiatric care.

While BNSS total scores systematically showed small but significant correlations with time use, the BNSS subdomains expressed a differential impact on NPA and PA in different settings. Interestingly, the avolition domain proved to be significantly associated with NPA in both settings, shifting the required emphasis on interventions such as behavioural activation^56^. Notably, together with demotivating beliefs and deficits of anticipatory pleasure^23^, avolition may still represent an overlooked therapeutic target^3^.

### More negative symptoms, more time spent in inactivity

Even if the conceptualisation of negative symptoms has been refined throughout time to reduce inappropriate overlap with non-specific behavioural and psychopathological facets (e.g., social withdrawal and depressive disorders)^3^, sedentary attitudes and reduced physical activity have long been acknowledged to be over-represented in patients with SSD^19^, and their association with negative symptom severity should not be overlooked. In this regard, this study highlighted a progressive increase of NPA at the expense of PA when moving from low- to high-levels of negative symptom severity, irrespective of treatment setting. The only dimension of time use characterised by remarkably different values in each subgroup was goal-oriented time use during work days, confirming the strong association of negative symptoms with the burden of disability in patients with SSDs in terms of reduced productivity^1,3^. To address this relevant area, recovery-oriented approaches should be considered that stress the importance of adequate and satisfactory personal and social functioning, which includes structured and valuable time use^57^.

### More time spent in inactivity, more negative daily emotions

Recently, the importance of negative beliefs and attitudinal factors in patients with SSDs has been emphasised^10^, enriching perspectives which address negative symptoms in light of neurocognitive deficits^24,27^. On the one hand, negative emotions may represent a stable feature in case of comorbidity in patients with SSDs and mood disorders, or the clinical expression of schizoaffective disorder^44^. Conversely, the daily experience of individuals suffering from major mental illness is greatly influenced by affective and emotional experiences^58^, which may be overlooked in a strictly categorical framework, despite the implications for the recovery process^26,29^. A more inclusive, psychologically-oriented conceptualisation of SSDs suggests that negative symptoms may be partly seen as a compensatory coping pattern of disengagement in response to psychotic symptoms, perceived interpersonal threat, and anticipated failure in various social tasks and activities^51,52,59^. In other words, specific beliefs and cognitive appraisals tied to negative emotional states may intervene in the daily experience of patients, accounting for a reduction of productive and goal-oriented activities^27,59^. In this light, the present study found a significant association of negative emotions with time spent in NPA, even when accounting for the role of negative symptom severity. In conclusion, the likely bidirectional relationship between emotions and time use could be seen as a detrimental interplay with drastic consequences on psychosocial functioning as well as the use and effectiveness of interventions^23^. The present results, therefore, enrich the literature available on the topic by introducing ESM as a source of information on patients’ emotions^30,31^, with potential therapeutic implications. With a higher burden of negative symptoms leading to a reduced impact of lifestyle interventions^22^, negative emotions may represent an important therapeutic target by addressing both the cognitive and emotional components of negative symptoms and the behavioural consequences on active lifestyle and patients’ ability to engage in PA (e.g., maintaining an occupation and/or investing time in vocational activities and leisure). In this framework, a recent study outlined that negative symptomatology responds to a combination of motivational interviewing and cognitive-behavioural therapy^60^ – indirectly corroborating the increasingly acknowledged role of negative emotions and cognitions in the clinical presentation of motivational negative symptoms^27,28,31,52^.

### Limitations

Several limitations of the present study must be acknowledged. First, all participants were assessed from October 2020 to October 2021, during the COVID-19 pandemic, which led to containment measures and changes in daily activities: this may partly limit the generalisability of the results. However, it should be highlighted that the containment measures did affect both patients and healthy controls: therefore, the differences found between the two groups hold true having the assessments been made in the same temporal period. Second, the categorisation of daily activities was based on previous literature, and it was refined to match the main goals of the research project. We therefore acknowledge that different groupings of daily activity categories could be used to study this area. Third, there were no detailed time frames (i.e., minutes) for each specific daily activity. Indeed, the TUS questionnaire included 24 columns (one for each hour of the day), and participants could perform and select more than one activity, so that the reported mean and standard deviation for daily activities corresponds to a “count” rounded to 60 minutes, without considering activities lasting fractions of an hour. Fourth, even if the TUS was completed in real time or at latest in the evenings, its intrinsic retrospective, self-reported nature may be associated with some degree of inaccuracy (e.g., the fact that it could be completed after a certain amount of time after which recalling daily activities may have become difficult, and the fact that the chosen working day may not necessarily be representative of a typical day). Moreover, we did not specifically study the psychometric properties of ESM items because they have been chosen from a consolidated repository of ESM items, where all items recommended for inclusion in ESM studied have been psychometrically validated. Further, the heterogeneity of the clinical sample – although representative of the real-world practice – may limit the validity of the findings in specific clinical populations. Moreover, due to absent information, we did not control for the co-occurrence of depressive symptoms, which may represent secondary negative symptoms^8^. To conclude, the cross-sectional nature of the study prohibits drawing conclusions about the directionality of the relationship between time use and clinical outcomes, and – more specifically – it does not allow to detect a putative causal role of negative emotions or symptoms with regard to time use. Future research should address these limitations in prospective studies.

## Conclusion

The present work contributes to highlighting the complex relationship between negative symptoms, time use, and negative emotional states in a large, real-world clinical sample. While the potentially broad and non-specific categories of negative symptoms and inactivity have frequently been addressed only in light of occupational therapy^34–36^, the outlined role of negative emotions – observed through ESM – may stimulate further investigations to inform and improve the planning and the effectiveness of psychosocial interventions for patients with SSDs targeting negative symptoms, daily activity, negative emotions and functionality in a recovery-oriented, person-centred approach^37,39^.

### Supplementary information

Supplementary Material

## Data Availability

Dataset referring to this manuscript is published with restricted access on Zenodo platform and accessible at this link: 10.5281/zenodo.6867888.
