# Supplementary material for: Interplay between negative symptoms, time spent doing nothing, and negative emotions in patients with schizophrenia spectrum disorders: results from a 37-site study
Source: Schizophrenia (Heidelb). 2023 Sep 21;9(1):63. doi: 10.1038/s41537-023-00372-x (PMC10514038; doi:10.1038/s41537-023-00372-x)
Supplement: Supplementary file 1 — Supplementary Material [file 41537_2023_372_MOESM1_ESM.docx]

**SUPPLEMENTARY MATERIAL**

**TABLE 1S.**

**Guideline list of activities for TUS, as classified by EUROSTAT**

|  | **ACTIVITY** | **ACTIVITY DESCRIPTION** |  |
| --- | --- | --- | --- |
|  | **Working** | *Work-related activities (and lunch breaks or breaks during work) or looking for work.* | |
|  | **Studying** | *Activities related to school or training, such as taking courses or lessons, taking exams, studying, doing homework.* | |
|  | **Doing housework** | *Activities related to the management of your home or personal property (such as a car), such as: cooking, washing dishes, laundry, tidying up, cleaning, sewing, ironing, building and renovating the house (e.g., painting the walls, repairing something in the house), shopping, buying services (e.g., electricity, gas), going to the hairdresser / barber, managing family life.* | |
|  | **Taking care of someone or something** | *Activities related to the care of one's family (adults or children), animals or plants: for example, helping or playing with children, providing physical care, or keeping company); gardening (care of plants, vegetable garden, flowers); taking care of animals (feeding, walking).* | |
|  | **Voluntary work** | *Voluntary work carried out within a group / association, or aid given free of charge to people from other families (including children not living together).* | |
|  | **Sleeping** | *Sleeping, light or deep sleep.* | |
|  | **Staying in bed due to feeling unwell** | *Awake but in bed due to feeling unwell.* | |
|  | **Resting, doing nothing** | *Activities of relaxation, resting, thinking, or meditating, doing nothing (without sleeping), smoking.* | |
|  | **Doing leisure activities** | *Leisure activities, such as going to the cinema, theatre, concert, exhibition or museum, taking cultural trips, painting, photographing, making videos, playing an instrument, writing poetry, making collections, using e-mail (not for work), search for information on the internet, playing (even on the PC or with video games, or with animals), reading (newspapers, books, magazines), socializing, making and receiving visits, sending text messages, conversing on the phone, celebrating, chatting with someone.* | |
|  | **Eating** | *Meal-related activities, such as eating, drinking, having lunch, dinner, drinking.* | |
|  | **Self-caring** | *Self-care activities, such as washing, dressing, shaving, taking medical care (e.g., having a medical examination, taking medicine).* | |
|  | **Doing physical activity** | *Leisure activities that require physical exercise such as sports, dancing, walking, strolling, running, playing with the ball, fitness, hunting, fishing, mushroom / plant picking, and all outdoor sports activities.* | |
|  | **Getting around** | *Time spent travelling on or in transport or by feet.* | |
|  | **Watching TV or listening to the radio** | *Leisure activities such as watching television, videos, TV series, movies, or listening to the radio / music.* | |
|  | **Participating to religious activities** | *Activities of participation in religious activities, such as religious meetings / gatherings or religious ceremonies, praying, going to a place of worship, going to the cemetery.* | |

**TABLE 2S.**

**DIFFERENCES IN TIME SPENT IN NON-PRODUCTIVE AND PRODUCTIVE ACTIVITIES BOTH DURING WORK DAYS AND DURING SUNDAYS FOR DIFFERENT NEGATIVE SYMPTOM SEVERITY TERTILES**

|  |  |  |  |  |  | **LNSS Vs MNSS** | | **LNSS Vs HNSS** | | **HNSS Vs MNSS** | | |
| --- | --- | --- | --- | --- | --- | --- | --- | --- | --- | --- | --- | --- |
|  | **Average activities counts (*SD*)** | **LNSS**  **(*n* = 210)** | **MNSS**  **(*n* = 206)** | **HNSS**  **(*n* = 202)** | ***p*** | **Mean Difference**  **(*CI* 95%)** | ***p*** | **Mean Difference**  **(*CI* 95%)** | ***p*** | **Mean Difference**  **(*CI* 95%)** | ***p*** |  |
| **NPA** | **Work days** | 4.3 (2.8) | 4.7 (2.9) | 5.2 (2.8) | **.005** | -.43 (-1.10; .24) | .363 | -.91 (-1.58; -.23) | **.004** | -.47 (-1.15; .20) | .277 |  |
|  | **Sundays** | 5.4 (3.1) | 5.6 (2.9) | 6.0 (2.8) | .134 | -.28 (-.98; .42) | .993 | -.59 (-1.29; .12) | .135 | -.31 (-1.01; .40) | .903 |  |
| **PA°** | **Work days** | 4.2 (3.3) | 3.3 (3.0) | 2.6 (2.6) | **<.001** | .86 (.15; 1.56) | **.011** | 1.57 (.86; 2.27) | **<.001** | .71 (.00; 1.42) | **.050** |  |
|  | **Sundays** | 2.7 (3.0) | 2.3 (2.6) | 1.9 (2.6) | **.018** | .35 (-.30; .99) | .600 | .77 (.12; 1.42) | .271 | .42 (-.23; 1.08) | .272 |  |

**Legend**: NPA, Non-Productive Activities; PA, Productive Activities; CI, confidence interval; SD, standard deviation; LNSS, Low Negative Symptom Severity; MNSS, Medium Negative Symptom Severity; HNSS, High Negative Symptom Severity. ***p*** adjusted for multiple comparisons using Bonferroni procedure; °, variable log-transformed before ANOVA.

**TABLE 3S.**

**ASSOCIATION OF BNSS AND NON-PRODUCTIVE ACTIVITIES WITH NEGATIVE EMOTIONS IN RESIDENTIAL PATIENTS AND OUTPATIENTS**

| **UNIVARIATE MODELS** | | | |
| --- | --- | --- | --- |
| **Work days** | | | |
| **Variable** | $\hat{\beta}$ | ***CI* (95%)** | ***p*** |
| BNSS total score | 0.178 | -0.016; 0.372 | .071 |
| **Sundays** | | | |
| **Variable** | $\hat{\beta}$ | ***CI* (95%)** | ***p*** |
| BNSS total score | 0.225 | 0.024; 0.426 | **.029** |
| **UNIVARIATE MODELS** | | | |
| **Work days** | | | |
| **Variables** | $\hat{\beta}$ | ***CI* (95%)** | ***p*** |
| Non-productive activities | 1.389 | 0.773; 2.006 | **<.001** |
| **Sundays** | | | |
| **Variables** | $\hat{\beta}$ | ***CI* (95%)** | ***p*** |
| Non-productive activities | 1.512 | 0.916; 2.108 | **<.001** |
| **UNIVARIATE MODELS** | | | |
| **Work days** | | | |
| **Variables** | $\hat{\beta}$ | ***CI* (95%)** | ***p*** |
| Poly-AP treatment | 2.195 | 0.035; 4.355 | **.046** |
| **Sundays** | | | |
| **Variables** | $\hat{\beta}$ | ***CI* (95%)** | ***p*** |
| Poly-AP treatment | 4.193 | 2.044; 6.341 | **<.001** |
| **MULTIVARIABLE MODELS (controlled for BNSS total score and Poly-AP treatment)** | | | |
| **Work days** | | | |
| **Variables** | $\hat{\beta}$ | ***CI* (95%)** | ***p*** |
| Non-productive activities | 1.681 | 0.607; 2.756 | **.002** |
| **Sundays** | | | |
| **Variables** | $\hat{\beta}$ | ***CI* (95%)** | ***p*** |
| Non-productive activities | 1.385 | 0.404; 2.365 | **.006** |

**Legend**: CI, confidence interval

**TABLE 4S.**

**ASSOCIATION OF NON-PRODUCTIVE ACTIVITIES WITH NEGATIVE EMOTIONS IN RESIDENTIAL PATIENTS, OUTPATIENTS AND HEALTHY CONTROLS**

|  | **Residential care patients**  **(*n* = 57)** | | **Outpatients**  **(*n* = 46)** | | **Healthy controls**  **(*n* = 112)** | |
| --- | --- | --- | --- | --- | --- | --- |
| **Work days** | | | | | | |
| **Variables** | $\hat{\beta}$ (*CI* 95%) | ***p*** | $\hat{\beta}$ (*CI* 95%) | ***p*** | $\hat{\beta}$ (*CI* 95%) | ***p*** |
| Non-productive activities | 2.081  (.740; 3.421) | **.003** | .774  (-.588; 2.136) | .259 | .694  (-.266; 1.654) | .155 |
| **Sundays** | | | | | | |
| **Variables** | $\hat{\beta}$ (*CI* 95%) | ***p*** | $\hat{\beta}$ (*CI* 95%) | ***p*** | $\hat{\beta}$ (*CI* 95%) | ***p*** |
| Non-productive activities | 2.017  (.508; 3.527) | **.010** | 1.225  (-.320; 2.769) | .118 | 1.262  (-.417; 2.940) | .139 |

**Legend**: CI, confidence interval.

**TABLE 5S.**

**SOCIO-DEMOGRAPHIC DATA, CLINICAL FEATURES AND TIME USE IN PARTICIPANTS THAT COMPLETED ESM WITH SCHIZOPHRENIA-SPECTRUM DISORDERS AND HEALTHY CONTROLS.**

|  |  |  |  |  | **Residential Care Vs Outpatients** | | **Residential Care Vs Controls** | | **Outpatients Vs Controls** | |
| --- | --- | --- | --- | --- | --- | --- | --- | --- | --- | --- |
| **Characteristic** | **Residential care patients**  **(*n* = 66)** | **Outpatients**  **(*n* = 55)** | **Healthy controls**  **(*n* = 112)** | ***p*** | **Mean Difference**  **(*CI* 95%)** | ***p*** | **Mean Difference**  **(*CI* 95%)** | ***p*** | **Mean Difference**  **(*CI* 95%)** | ***p*** |
| **SOCIO-DEMOGRAPHIC INFORMATION** | | | | | | | | | | |
| **Age (*M* ± *SD*)** | 42.8 (10.4) | 39.0 (10.6) | 41.4 (10.2) | .132 | - | - | - | - | - | - |
| **Sex (n males, %)** | 47 (71.2%) | 30 (54.5%) | 67 (59.8%) | .143 | - | - | - | - | - | - |
| **Marital status (*n*, %)** |  |  |  |  |  |  |  |  |  |  |
| *Divorced/widowed* | 7 (10.6%) | 6 (10.9%) | 6 (5.4%) | **<.001** | **-** | .952 | **-** | **<.001** | **-** | **<.001** |
| *Married/cohabiting* | 5 (7.6%) | 5 (9.1%) | 78 (69.6%) |  |  |  |  |  |  |  |
| *Single* | 54 (81.8%) | 44 (80.0%) | 28 (25.0%) |  |  |  |  |  |  |  |
| **Education years (*M* ± *SD*)** | 11.5 (3.4) | 12.6 (2.4) | 16.5 (4.9) | **<.001** | -1.1  (-.7; 2.8) | .307 | -5.1  (-6.5; -3.6) | **<.001** | -4.0  (-5.5; -2.4) | **<.001** |
| **Working status (*n*, %)** |  |  |  |  |  |  |  |  |  |  |
| *Working* | 11 (16.7%) | 26 (47.3%) | 103 (92.0%) | **<.001** | **-** | **<.001** | **-** | **<.001** | **-** | **<.001** |
| *Studying* | 3 (4.5%) | 7 (12.7%) | 8 (7.1%) |  |  |  |  |  |  |  |
| *Not working nor studying* | 52 (78.8%) | 22 (40.0%) | 1 (0.9%) |  |  |  |  |  |  |  |
| **Body Mass Index (*M* ± *SD*)** | 26.1 (4.9) | 29.3 (6.5) | 24.2 (3.7) | **<.001** | -3.2  (-5.3; -1.1) | **.001** | 1.9  (.2; 3.7) | **.028** | 5.1  (3.2; 7.0) | **<.001** |
| **PSYCHOPATHOLOGY** | | | | | | | | | | |
| **SSD diagnosis (*n*, %)** |  |  |  |  |  |  |  |  |  |  |
| *Delusional Disorder* | 3 (4.5%) | 3 (5.5%) | - | .996 | - | - | - | - | - | - |
| *Schizophreniform Disorder* | 1 (1.5%) | 1 (1.8%) | - |  |  |  |  |  |  |  |
| *Schizophrenia* | 37 (56.1%) | 32 (58.2%) | - |  |  |  |  |  |  |  |
| *Schizoaffective Disorder* | 17 (25.8%) | 13 (23.6%) | - |  |  |  |  |  |  |  |
| *Other SSD (i.e., without specification, with other specification)* | 8 (12.1%) | 6 (10.9%) | - |  |  |  |  |  |  |  |
| **Psychiatric comorbidities (*n* yes, %)** | 30 (45.5%) | 12 (21.8%) | - | **.007** | - | - | - | - | - | - |
| **Illness duration (years, *M* ± *SD*)** | 19.4 (10.3) | 15.9 (8.9) | - | .053 | - | - | - | - | - | - |
| **Antipsychotic drugs (*M* ± *SD*)** | 1.7 (0.8) | 1.5 (0.8) | - | .220 | - | - | - | - | - | - |
| **Non-antipsychotic psychotropic drugs (*M* ± *SD*)** | 1.7 (1.3) | 0.9 (0.8) | - | **<.001** | .8  (.4; 1.1) | - | - | - | - | - |
| *Antidepressants* | 0.3 (0.5) | 0.4 (0.5) | - | .199 | - | - | - | - | - | - |
| *Benzodiazepines* | 1.0 (0.9) | 0.4 (0.5) | - | **<.001** | .6  (.3; .9) | - | - | - | - | - |
| *Mood stabilisers* | 0.4 (0.6) | 0.1 (0.3) | - | **<.001** | .3  (.1; .5) | - | - | - | - | - |
| **BNSS total score***°* **(*M* ± *SD*)** | 23.4 (14.5) | 16.8 (13.4) | - | **.011** | 6.6  (1.5; 11.6) | - | - | - | - | - |
| *Anhedonia* | 1.9 (1.4) | 1.4 (1.4) | - | **.039** | .5  (.1; 1.1) | ~~-~~ | ~~-~~ | ~~-~~ | ~~-~~ | ~~-~~ |
| *Distress°* | 1.6 (1.5) | 1.0 (1.3) | - | **.020** | .6  (.1; 1.1) | - | - | - | - | - |
| *Asociality* | 2.0 (1.4) | 1.8 (1.4) | - | .323 | .3  (-.2; .7) | - | - | - | - | - |
| *Avolition* | 2.1 (1.5) | 1.6 (1.4) | - | .062 | .5  (-.1; 1.1) | - | - | - | - | - |
| *Blunted affect°* | 1.7 (1.4) | 1.2 (1.4) | - | **.032** | .6  (.1; 1.1) | - | - | - | - | - |
| *Alogia°* | 1.3 (1.5) | 0.8 (1.1) | - | **.025** | .6  (.1; 1.0) | - | - | - | - | - |
| **DAILY ACTIVITIES FROM TIME USE SURVEY (COUNT FREQUENCY)** | | | | | | | | | | |
| *NPA on Sundays (M±SD)*  *Median (range)* | 6.0 (2.8)  5 (0-14) | 5.6 (3.0)  5 (1-16) | 3.1 (2.0)  3 (0-10) | **<.001** | .4  (-.7; 1.5) | .618 | 2.9  (2.0; 3.9) | **<.001** | 2.5  (-3.5; -1.5) | **<.001** |
| *NPA on work days (M±SD)*  *Median (range)* | 5.2 (2.6)  4 (1-11) | 4.4 (2.5)  4 (1-10) | 1.1 (1.2)  1 (0-7) | **<.001** | .7  (-.1; 1.6) | .127 | 4.1  (3.3; 4.8) | **<.001** | 3.3  (2.6; 4.1) | **<.001** |
| *PA on Sundays (M±SD)°*  *Median (range)* | 1.6 (1.7)  1 (0-6) | 3.3 (3.5)  2 (0-18) | 5.3 (4.2)  4 (0-17) | **<.001** | -1.8  (-3.3; -.3) | **.016** | -3.7  (-5.0; -2.4) | **<.001** | -2.0  (-3.3; -.6) | **.002** |
| *PA on work days (M±SD)°*  *Median (range)* | 2.3 (2.1)  2 (0-8) | 5.4 (3.2)  4 (0-12) | 11.1 (3.8)  11 (0-28) | **<.001** | -3.0  (-4.4; -1.7) | **<.001** | -8.8  (-9.9; -7.6) | **<.001** | -5.7  (-7.0; -4.5) | **<.001** |

**Legend**: M ± SD, mean ± standard deviation; SSD, Schizophrenia Spectrum Disorder; BNSS, Brief Negative Symptom Scale; NPA, Non-Productive activities; PA, Productive Activities. *, *p* computed using Monte Carlo-simulation (B = 2000); °, variable modelled on log scale.

**TABLE 6S.**

**STATISTICS OF TESTS REPORTED IN TABLE 1.**

| **Characteristic** | *Statistics* |
| --- | --- |
| **Age^1^** | .431 |
| **Sex^2^** | 4.114 |
| **Marital status^2^** | 278.933 |
| **Education years^1^** | 97.277 |
| **Working status^2^** | 257.819 |
| **Body Mass Index^1^** | 30.376 |
| **SSD diagnosis^2^** | 11.643 |
| **Psychiatric comorbidities^2^** | 17.446 |
| **Illness duration^3^** | .236 |
| **Antipsychotic drugs^3^** | 5.754 |
| **Non-antipsychotic psychotropic drugs^3^** | 6.915 |
| *Antidepressants***^3^** | .807 |
| *Benzodiazepines***^3^** | 9.069 |
| *Mood stabilisers***^3^** | 3.556 |
| **BNSS total score^3^** | 4.482 |
| *Anhedonia***^3^** | 4.555 |
| *Distress***^3^** | 4.442 |
| *Asociality***^3^** | 2.862 |
| *Avolition***^3^** | 3.596 |
| *Blunted affect***^3^** | 4.751 |
| *Alogia***^3^** | 2.610 |
| *NPA on Sundays***^1^** | 38.926 |
| *NPA on work days***^1^** | 89.102 |
| *PA on Sundays***^1^** | 29.938 |
| *PA on work days***^1^** | 170.941 |

**Reported statistics: ^1^: t, ^2^: chi-square, ^3^: F**

**TABLE 7S.**

**STATISTICS OF TESTS REPORTED IN TABLE 2S.**

|  | **Average activities counts** | ***F-Statistic*** |
| --- | --- | --- |
| **NPA** | **Work days** | 5.248 |
|  | **Sundays** | 2.015 |
| **PA** | **Work days** | 14.169 |
|  | **Sundays** | 4.042 |

**TABLE 8S.**

**STATISTICS OF TESTS REPORTED IN TABLE 3S.**

| **Variable** | **F-Statistic** |
| --- | --- |
| **UNIVARIATE MODELS** | |
| **Work days** | |
| BNSS total score | 3.309 |
| **Sundays** | |
| BNSS total score | 4.919 |
| **Work days** | |
| Non-productive activities | 19.723 |
| **Sundays** | |
| Non-productive activities | 24.966 |
| **Work days** | |
| Poly-AP treatment | 4.010 |
| **Sundays** | |
| Poly-AP treatment | 14.788 |
| **MULTIVARIABLE MODELS (controlled for BNSS total score and Poly-AP treatment)** | |
| **Work days** | |
| Non-productive activities | 4.436 |
| **Sundays** | |
| Non-productive activities | 4.476 |

**TABLE 9S.**

**STATISTICS OF TESTS REPORTED IN TABLE 4S.**

|  | **Residential care patients** | **Outpatients** | **Healthy controls** |
| --- | --- | --- | --- |
| **Work days** | | | |
| **Variables** | F-Statistic | F-Statistic | F-Statistic |
| Non-productive activities | 9.635 | 1.306 | 2.052 |
| **Sundays** | | | |
| Non-productive activities | 7.127 | 2.530 | 2.219 |

**TABLE 10S.**

**STATISTICS OF TESTS REPORTED IN TABLE 5S.**

| **Characteristic** | *Statistics* |
| --- | --- |
| **Age^1^** | 2.921 |
| **Sex^2^** | 3.370 |
| **Marital status^2^** | 89.563 |
| **Education years^1^** | 36.732 |
| **Working status^2^** | 124.419 |
| **Body Mass Index^1^** | 25.323 |
| **SSD diagnosis^2^** | 11.643 |
| **Psychiatric comorbidities^2^** | 7.463 |
| **Illness duration^3^** | 2.144 |
| **Antipsychotic drugs^3^** | .743 |
| **Non-antipsychotic psychotropic drugs^3^** | 4.127 |
| *Antidepressants***^3^** | 1.115 |
| *Benzodiazepines***^3^** | 4.389 |
| *Mood stabilisers***^3^** | 3.680 |
| **BNSS total score^3^** | 2.185 |
| *Anhedonia***^3^** | 2.246 |
| *Distress***^3^** | 2.034 |
| *Asociality***^3^** | .867 |
| *Avolition***^3^** | 1.802 |
| *Blunted affect***^3^** | 1.927 |
| *Alogia***^3^** | .377 |
| *NPA on Sundays***^1^** | 33.967 |
| *NPA on work days***^1^** | 99.939 |
| *PA on Sundays***^1^** | 14.132 |
| *PA on work days***^1^** | 129.674 |

**Reported statistics: ^1^: t, ^2^: chi-square, ^3^: F**
